# Dormancy in Breast Cancer, the Role of Autophagy, lncRNAs, miRNAs and Exosomes

**DOI:** 10.3390/ijms23095271

**Published:** 2022-05-09

**Authors:** Leila Jahangiri, Tala Ishola

**Affiliations:** 1 Division of Cellular and Molecular Pathology, Department of Pathology, Addenbrookes Hospital, University of Cambridge, Cambridge CB2 0QQ, UK; 2 Department of Life Sciences, Birmingham City University, Birmingham B15 3TN, UK; 3 School of Science & Technology, Nottingham Trent University, Clifton Lane, Nottingham NG11 8NS, UK

**Keywords:** dormancy, breast cancer, autophagy, lncRNAs, miRNAs, exosomes, treatment

## Abstract

Breast cancer (BC) is the most frequently diagnosed cancer in women for which numerous diagnostic and therapeutic options have been developed. Namely, the targeted treatment of BC, for the most part, relies on the expression of growth factors and hormone receptors by these cancer cells. Despite this, close to 30% of BC patients may experience relapse due to the presence of minimal residual disease (MRD) consisting of surviving disseminated tumour cells (DTCs) from the primary tumour which can colonise a secondary site. This can lead to either detectable metastasis or DTCs entering a dormant state for a prolonged period where they are undetectable. In the latter, cells can re-emerge from their dormant state due to intrinsic and microenvironmental cues leading to relapse and metastatic outgrowth. Pre- and clinical studies propose that targeting dormant DTCs may inhibit metastasis, but the choice between keeping them dormant or forcing their “awakening” is still controversial. This review will focus on cancer cells’ microenvironmental cues and metabolic and molecular properties, which lead to dormancy, relapse, and metastatic latency in BC. Furthermore, we will focus on the role of autophagy, long non-coding RNAs (lncRNAs), miRNAs, and exosomes in influencing the induction of dormancy and awakening of dormant BC cells. In addition, we have analysed BC treatment from a viewpoint of autophagy, lncRNAs, miRNAs, and exosomes. We propose the targeted modulation of these processes and molecules as modern aspects of precision medicine for BC treatment, improving both novel and traditional BC treatment options. Understanding these pathways and processes may ultimately improve BC patient prognosis, patient survival, and treatment response.

## 1. The Molecular Characteristics of Dormant Breast Cancer Cells

Circa 1 in 8 women will be diagnosed with breast cancer (BC) within the duration of their life [1]. This accounts for close to a quarter of all cancer cases and approximately 16% of cancer-related mortality in the western world; hence, it is regarded as a major health concern in women [2,3]. BC is immensely heterogeneous with diverse histopathological properties and molecular profiles. From a molecular viewpoint, 4 distinct BC subtypes have been identified including luminal A, luminal B, human epidermal growth factor receptor 2 positive (HER2+), and triple-negative [4,5]. In addition, BC can be categorised into immunohistological subtypes based on oestrogen receptor (ER), progesterone receptor (PR), and human epidermal growth factor receptor 2 (HER2) status. For example, luminal A cases are often ER+ and/or PR+, HER2−, while luminal B are usually ER+ and/or PR+, HER2+/−. HER2-enriched are ER−, PR−, HER2+, while triple-negative/basal-like are ER−, PR−, HER2− [6,7]. Accordingly, at the time of diagnosis, BC can be categorised into 4 main stages including in situ (0), early-stage invasive (I, IIa, IIb), locally advanced (IIIa, IIIb, IIIc), and metastatic (IV), with 5-year survival percentages of 100, 98, 57 and 23.4, respectively [1]. In other words, the 5-year survival of non-invasive, regional invasive and highly invasive BC is 91%, 86%, and 30%, respectively [4].

**Figure 1:**
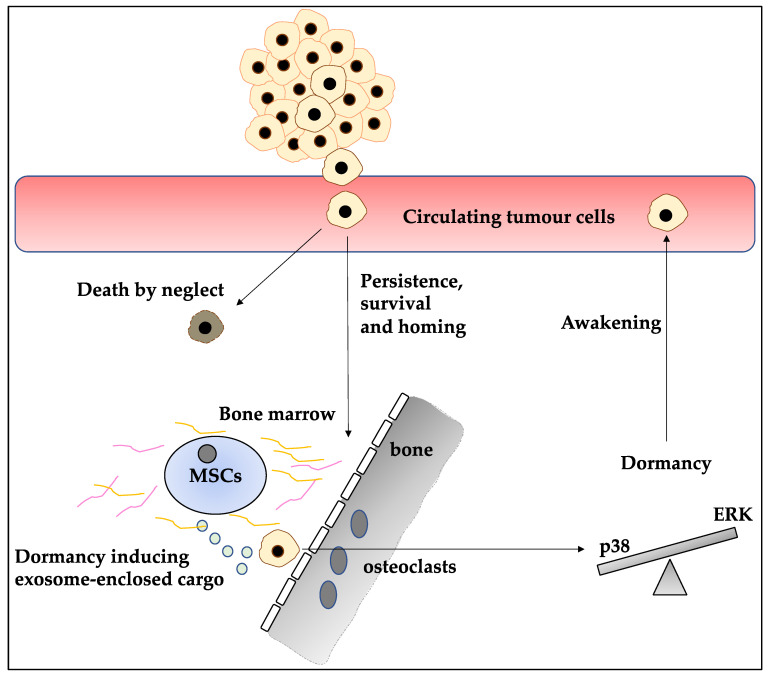
The life cycle of tumour cells from the primary tumour to dormancy in BC. Tumour cells exiting the primary tumour site by digesting the extracellular matrix proteins and downregulating E-cadherin, can enter the bloodstream and constitute circulating tumour cells (CTCs). The pool of CTCs is large, and the majority will undergo cell death while a fraction of these cells may persist, survive, and disseminate to, for example, the bone marrow. Upon exposure to dormancy inducing cargo enclosed in exosomes generated by, for instance, the bone marrow mesenchymal stem cells (BM-MSCs), tumour cells may enter the state of dormancy marked by a high ratio of p38 MAPK/ERK. The dormant cell may be awakened by various mechanisms and re-entre the blood.

Concerning treatment, stage 0 (in situ) can increase the risk of subsequent invasive BC and can be lobular or ductal with no treatment/tamoxifen prophylaxis, and surgery/radiotherapy recommended as primary treatment options, respectively [1]. In early-stage invasive (I and II), surgery and radiation therapy are recommended [8,9], while in stage 3, chemotherapy, surgery and radiotherapy have been suggested [10,11,12,13,14]. In stage IV, radiation therapy and symptom treatment are advised. Notably, adjuvant therapy can be administered starting from the early-stage invasive (I and II) stage and this will depend on the presence of hormone receptors and HER2 overexpression. For instance, in early-stage invasive (I and II) if the patient is hormone receptor-negative, positive or overexpresses HER2, chemotherapy, chemotherapy/endocrine therapy, and chemotherapy/trastuzumab treatment is recommended, respectively [2,15]. Despite the use of these treatment options, BC survivors can suffer disease recurrence due to the presence of minimal residual disease (MRD). MRD including disseminated tumour cells (DTCs) homed in the bone marrow can re-enter the circulation and invade other organs such as the lungs [16]. It is, however, important to understand the current definitions of the terminology. For example, circulating tumour cells (CTCs) represent a large pool of tumour cells entering the circulation, while only a fraction of them, DTCs, can persist and enter metastatic sites and in turn, only a fraction of DTCs can lead to metastasis [17] (Figure 1).

On the other hand, our understanding of the properties of dormant cells or resistant dormant cells is vastly limited. Nonetheless, by definition, resistant dormant cells represent tumour cells which may be resistant to therapy with non- or slow-proliferation capacity and hence may play a role in disease relapse. These cells may comprise two subpopulations; quiescent dormant tumour cells, which are also slow-cycling/slow-proliferating or stalled reversibly in the G0 phase of the cell cycle, and senescent dormant tumour cells that have irreversibly exited the cell cycle. The cell population intended throughout this review, however, encapsulates the quiescent dormant tumour cells described above, referred to herein as “dormant cells” that may not respond to therapy and subsequently lead to metastasis and disease recurrence [18,19,20,21,22,23,24]. In addition, the literature reports overlapping characteristics of cancer stem cells (CSCs) and quiescent dormant tumour cells, for instance, the ability to resist therapy, although in our opinion, cancer persister cells may be a better representative of the cell population resisting therapy, while CSCs may represent the cell population propagating an existing tumour [25]. Nonetheless, CSCs may be viewed as dormancy-competent tumour repopulating cells [26,27].

From a clinical viewpoint, the presence of DTCs in the bone marrow in BC patients is associated with a higher risk of local and distal recurrence and reduced survival [28,29]. This dissemination may occur in the early or late stages of the disease, consistent with the premise that cancer dissemination can precede primary cancer detection essentially shedding light on the “cancer without disease” notion [30]. Overall, distant recurrence of BC due to dormant cells could be viewed as a significant clinical challenge; hence, novel therapeutic strategies could aim to retain these cells in a dormant state, eliminate them or prevent their metastatic outgrowth [31].

Moreover, intrinsic and extrinsic factors and related processes including autophagy and molecules including lncRNAs and micro RNAs (miRNAs) can influence dormancy. Autophagy, a multi-faceted pro-survival mechanism, comprises the destruction and recycling of cellular components to meet its energetic and metabolic requirements [32]. This mechanism can be activated in response to various cellular stresses, including hypoxia and starvation, although autophagy is active at a basal level in cells [33]. Changes to cellular and molecular processes including autophagic pathways may influence oncogenic processes, including dormancy and therapy resistance. For instance, dormant cells utilise autophagic processes to survive hypoxic and nutrient deficient tumour microenvironments [34,35,36,37,38,39,40,41]. Additionally, close to 40% of ER+ BC patient cases develop treatment resistance which may be due to autophagy; this is despite anti-ER treatment, including compounds directly inhibiting oestrogen binding or ER synthesis and function, being effective [42]. Furthermore, lncRNAs have been linked to tumourigenesis and tumour suppression [43], while exosome-encapsulated miRNAs can also influence cancer-related processes including dormancy [44,45,46]. Given this context, throughout this review we will focus on dormancy in BC, the role of autophagy in dormant cells and the interactions of these cells with lncRNAs and miRNAs. We discuss these processes and propose potential treatment options that may enhance the efficacy of current BC treatment regimens and patient quality of life.

## 2. Dormancy in BC Cells

MRD present in a dormant state can reflect dormancy in two categories inclusive of cellular or tumour mass level [20]. The former describes the state in which the tumour cells reversibly exit the cell cycle and enter a quiescent state induced by repressive microenvironmental or intrinsic signals as alluded to above. For example, dormancy on a cellular level can be induced by the activation of the p38 MAPK pathway, leading to a high ratio of the p38 MAPK/ERK signalling [24]. Notably, p38 is involved in stress response and cell cycle arrest [47], while the requirement of TGFβ2 (and TGFβ-RIII) for activation of MAPK p38 α/β and the resulting elevated ratio of the p38 MAPK/ERK signalling pathway has been suggested [48]. In addition, the role of the lysophosphatidic acid receptor (EDG2) in inducing an enhanced ratio of the p38 MAPK/ERK in BC has been reported [49].

**Figure 2:**
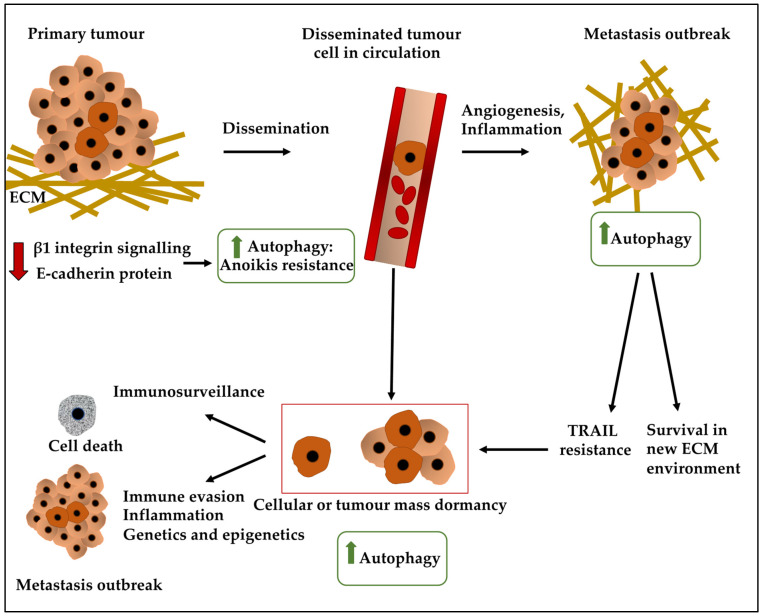
The lifecycle of a disseminated tumour cell from the primary tumour to metastasis. Primary tumour cells may reduce β1 integrin and E-cadherin levels, leading to increased autophagy and anoikis resistance. These cells, which break away from the primary tumour, can enter the circulation and eventually home in a secondary organ. A DTC in the circulation can take different routes hereafter, for instance, it can give rise to a secondary tumour and increase autophagy levels. This provides the cell(s) with an advantage to better survive the new ECM microenvironment and potentially gain resistance to cytotoxic agents such as TRAIL. Alternatively, DTC in circulation can home in a secondary site and enter a dormant state and undergo G0/G1 arrest or maintain a growth/death steady state in a dormant tumour mass, under the influence of microenvironmental factors or intrinsic cues. Immunosurveillance can lead to the elimination of the dormant cells, while immune evasion, inflammatory states and genetic/epigenetic factors may influence the escape of these cells from dormancy and the development of a metastatic outbreak in future years.

Contrastingly, tumour mass dormancy refers to the population of cancer cells that fail to give rise to a macroscopic tumour (greater than 2 mm) due to the balance between proliferation and apoptosis (Figure 2). This equilibrium is influenced by immunosurveillance, vascularisation, and the stroma [20,50,51,52]. Accordingly, immunosurveillance or indeed intense immune response regulated DTC immune clearance, a process that counteracts the proliferation of the tumour. One type of immune cell which may instigate a response through the recognition of a major histocompatibility complex (MHC) class 1-presented BC tumour antigen may be cytotoxic CD8+ T cells. Accordingly, immune evasion may occur, for instance through mechanisms such as loss of MHC class I, TAP proteins, and tumour-associated antigens [53]. This mechanism may be mediated by NLRC5, a transcriptional transactivator, although these processes may indeed be reversible upon the re-entry of the cell to the cell cycle [53]. For example, the upregulation of NLRC5 in quiescent cells could lead to the restoration of the MHC class 1 [53]. Although this study examined the downregulation of MHC class 1 in hair follicular stem cells in a quiescent state, this may be a feature which is applicable to other quiescent cells [53].

Furthermore, the angiogenic switch promoted tumour growth [20,50,51] and the extent to which vascularisation is present in the microenvironment and the resulting oxygen supply will impact cell survival, metabolism and metastatic behaviour [54]. The angiogenic switch, by definition, refers to the progression toward an angiogenic phenotype including steps involving the recruitment of new vasculature. This process converts non-angiogenic tumour lesions to angiogenic tumours now capable of growing beyond a microscopic mass [55]. The process of angiogenesis, however, can be suppressed during angiogenic dormancy due to the downregulation of pro-angiogenesis factors and the production of suppressors of angiogenesis, which may be mediated by genetic, epigenetic and microenvironmental cues [56,57]. As a result of angiogenic dormancy and the dominance of factors suppressing angiogenesis, the tumour will remain deprived of nutrients and oxygen due to insufficient vascularisation. This population-level dormancy is, hence, due to factors that counteract tumour proliferation. The source of these anti-angiogenic factors may be, for instance, endothelial cells that produce thrombospondin-1 (TSP1), leading to a growth/apoptosis balance and the hindrance of metastatic outbreak [56,57,58]. In addition, angiomotin and tropomyosin (the binding proteins of angiostatin and endostatin, respectively) may mediate anti-angiogenic activities in dormant tumour cells, while Eph receptor 5 (EphA5), a receptor tyrosine kinase involved in angiogenesis, may be linked to dormancy [56]. Accordingly, Almog and colleagues, reported that angiomotin and tropomyosin were upregulated in dormant tumour cells, while EphA5 was increased in the blood of mice with dormant tumour cells. Although this study was conducted in gliomas and glioblastomas, these mechanisms may indeed be relevant to BC [56]. Contrastingly, factors such as TGFβ1 and periostin may be viewed as tumour-promoting factors [57]. Collectively, BC dormant cells may have a distinct signature of angiogenic and anti-angiogenic players.

The bone marrow represents a niche for BC cell homing and comprises resident haemopoietic cells, endothelial cells, and mesenchymal stem cells in addition to endosteal and perivascular regions, among other components [57,59]. Notably, of the various BC subtypes, ER+ BC DTCs showed a greater tendency for bone marrow homing, while these DTCs may indeed be detected using EpCAM and pan-cytokeratin [60]. The DTCs that have subsequently homed in the bone marrow may receive intrinsic cues relevant to dormancy. Furthermore, local microenvironmental niches and their repertoire of secreted factors and extracellular matrix (ECM) proteins may induce dormancy in these DTCs [54]. For example, the basement membrane and crucially, laminin-111, may indeed facilitate dormancy, survival and resistance to treatment of BC DTCs [57,61]. Moreover, FGF-2, a peptide growth factor, can inhibit growth and proliferation by inducing p21, leading to the G1 cyclin complex inactivation [62,63], while α5β1-fibronectin contributed to the survival of the FGF2-responsive BC cells [64]. Consistently, the leukaemia inhibitor factor (LIF) produced a pro-dormancy signal to BC cells in the bone marrow. Interestingly, the loss of STAT3 and LIFR via the LIFR/STAT3/SOCS3 axes in BC cells reduced dormancy and CSC markers and promoted proliferation and colonisation [65]. Additionally, microenvironmental factors and signalling molecules such as mTORC1/mTORC2, TGFβ2, BMP, NR2F1, basic helix-loop-helix protein 3 (BHLHB3 or DEC2) may also induce dormancy [48,66,67,68]. Finally, the presence of endothelial cells (ECs), mesenchymal stem cells and osteoblastic niches in the bone marrow may also be significant [51,69]. Accordingly, BC cells can interact with E-selectin+ ECs thereby facilitating their homing and the maintenance of their dormant state [57].

Other factors which may affect dormancy could be epigenetic changes. For example, there may be a network of p38 dormancy-inducing signature genes that are epigenetic regulators including *NR2F1*, *TGFβ2*, and DNA methyltransferase 1 (*DNMT1*). For example, *NR2F1* may be linked to a repressive chromatin signature, while *TGFβ2* may be involved in chromatin remodelling [70,71,72]. In addition, a study revealed that treating BC cells with 5-azacytidine, an inhibitor of DNA methylation, may lead to reduced expression of genes such as *DNMT1*, which were also reduced in dormant BC cells. Consistently, genes which were usually upregulated in response to dormancy including *CDKN1A* were upregulated following treatment with 5-azacytidine [70,73,74]. This result highlighted the notion that epigenetic regulation of essential dormancy genes may indeed be significant.

Moreover, a study by Almog and colleagues has identified other potential markers of dormant cells which may be involved in epigenetic processes. For instance, they reported the role of H2BK, a protein involved in the nucleosome core, may be linked to tumour dormancy, albeit in glioblastoma cells [56]. Collectively, these studies drive the premise that many processes that are involved in the induction of dormancy may indeed be epigenetically modulated.

The processes that steer DTCs into dormancy or may lead to their awakening have been investigated and reviewed in Table 1.

Furthermore, studies have employed in vitro and in vivo models to understand these mechanisms in BC. For example, Ruth and colleagues revealed human BC xenograft exhibited dormancy following triple anti-HER2 (lapatinib, trastuzumab and pertuzumab) and anti-ER targeted therapy [20]. Briefly, GFP-labelled BT474-M1 BC cells were orthotopically xenografted to NOD-SCID immunodeficient mice leading to primary tumour growth. Following the mentioned targeted therapy, the primary tumour regressed to a non-palpable state, while residual BT474-M1 lesions, contained GFP+ cells with a very low Ki-67 proliferation index (2% as opposed to 25% for the injected primary tumour cells) [20]. A thorough inspection of these lesions revealed the presence of fibronectin and collagen type I in addition to vasculature expressing CD31 surface marker [20]. Furthermore, in this study, transgenic mice including *MTB*; *TetO-HER2/neu* (for example, eGFP-expressing *HER2/neu-Prim1*) formed primary mammary adenocarcinomas following doxycycline treatment [20]. Harvesting these tumours and their subsequent orthotopic xenografting to primary mammary fat pads of immunocompromised *nu/nu* mice maintained on doxycycline, led to tumour formation, while the withdrawal of doxycycline led to tumour regression. Notably, 28- and 56-days following doxycycline withdrawal, the remaining lesions displayed fibronectin and collagen type I abundance and a 40-fold reduced Ki-67 proliferation rate compared to primary tumour cells [20]. Moreover, the residual lesions displayed CD31+ rich vascularisation. This study ultimately revealed that dormant BC cells displayed low Ki-67, while fibronectin, collagen type I and CD31+ vasculature were enriched in the TME of these cells [20]. The study not only underscored the in vivo characteristics of dormant cells but also reiterated their markers and the properties of their surrounding tumour microenvironment. Consistently, in our opinion, sustaining the tumour cells in a state of dormancy may indeed prevent tumour relapse and tumour regrowth, a strategy that may indeed bear therapeutic value [95]. Chronic inflammation on the other hand supports the process of angiogenesis that can promote the growth and awakening of dormant cells [75]. Furthermore, long-term exposure to tobacco smoke triggering chronic inflammation can also lead to dormant cell awakening through neutrophil extracellular traps (NETs). Mechanistically, the NET-associated enzymes could digest laminin and trigger proliferation [79]. The remodelling of the ECM and signalling cascades converging on ERK can also lead to the awakening of dormant cells, a process that can trigger disease recurrence [76,77,78].

## 3. The Role of the Molecular Process of Autophagy in BC Dormancy and Treatment

### 3.1. The Role of Autophagy and Autophagy-Related Proteins and Players in BC

Macroautophagy (henceforth, autophagy), a catabolic cell-survival process marked by the lysosomal degradation mechanism of cellular components and organelles, is an adaptative response to various cellular stress including DNA damage, hypoxia, nutrient and trophic factor signal depletion, and chemical injury [32,33]. Accordingly, isolated double-membrane autophagosomes containing cellular components, organelles and fractions of the cytosol fuse with lysosomes, leading to the hydrolysis of these components. As a result, autophagy exerts a cytoprotective role by eliminating the damaged or defective proteins, organelle or cytosolic components and thereby can also supply cells with energy and nutrients [32,33].

**Figure 3:**
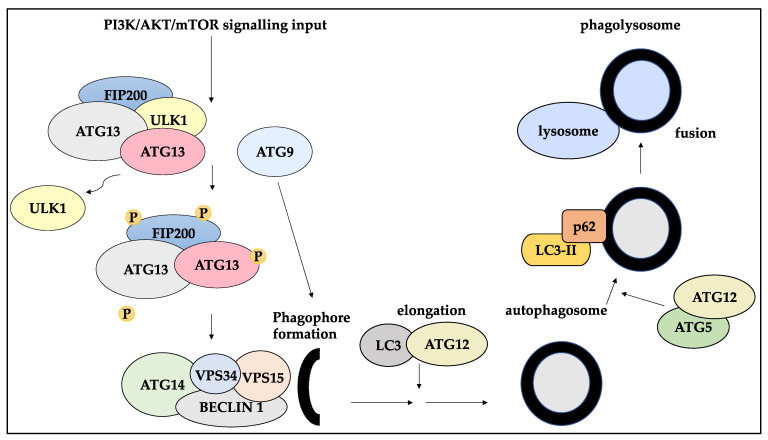
The molecular process of autophagy. In the autophagosome initiation processes, ULK1 is released from the ULK1/ATG13/FIP200 complex through mTOR-related mechanisms. This complex undergoes phosphorylation as ATG9 localises to the phagophore. These processes lead to the formation of the Beclin-1/ATG14L/VPS34/VPS15 complex which coats the isolation membrane. Subsequently cleaved LC3 and activated ATG12 contribute to the phagophore elongation process. Furthermore, ATG5/ATG12 mediate the coating of the autophagosome by p62/SQDTM1 bound LC3-II. The autophagosome then fuses with a lysosome to form a phagolysosome.

Key players of autophagy comprise over 30 autophagy-related (ATGs) genes. These genes are implicated in the process of autophagy, including the autophagosome initiation step, in which ULK1/ATG13/FIP200 released ULK1 in an mTOR-mediated manner [96]. Subsequently, the autophosphorylation of this complex is linked to the addition of ATG9 to the phagophore. Further, an isolation membrane coated by Beclin-1/ATG14L/VPS34/VPS15 complex is formed. The autophagosome elongation step is mediated by LC3 and ATG12, followed by cleaving and activation of each molecule, respectively. Then, ATG5/ATG12 facilitate the coating of the autophagosome by p62/SQDTM1 bound LC3-II. The autophagosome then fuses with a lysosome [96,97] (Figure 3).

Autophagy has been implicated in many cancers, and numerous studies have attempted to profile the function of various autophagic proteins in BC. For instance, the deletion of Beclin-1 (BECN1), a tumour suppressor protein essential for autophagy induction can promote pro-tumourigenic processes [98]. On the other hand, the expression of Beclin-1 was associated with ER+ BC and correlated with a favourable patient overall survival (OS) [99,100]. Consistently, Beclin-1 may be downregulated in HER2+ and triple-negative (basal-like) BC [101]. In addition, the downregulation of ULK1 in BC patients was shown to be an adverse prognostic predictor [102].

Furthermore, studies investigated BC subtype-specific autophagic gene expression patterns. For example, ATG5, and ATG3 were expressed in triple-negative breast cancer (TNBC) and promoted cell migration and favoured tumourigenesis, and the upregulation of LC3B correlated with reduced OS in BC patients [99,100,103].

Moreover, the role of ATG4A in the development of resistance to tamoxifen in BC was investigated. In evidence, ATG4A expression in the tumour tissue was correlated with reduced disease-free survival.

The PI3K autophagy inhibitor, 3-methyladenine, reduced ATG4A expression, while 4-hydroxytamoxifen increased ATG4A levels of MCF-7 BC cells. The knockdown of ATG4A levels following 4-hydroxytamoxifen treatment led to increased cell death by apoptosis. Collectively, this study revealed that the knockdown of ATG4A leads to autophagy suppression, and increased the sensitivity of BC cells to 4-hydroxytamoxifen [104]. In our opinion, autophagy genes including BECN1, ULK1 and ATGs influence BC tumourigenic processes, hence could be strategically modulated in a context-dependent manner.

### 3.2. Autophagy and Dormancy

Many pre-clinical and clinical studies have revealed that autophagy has a context-dependent function in regulating tumourigenesis. In the early stages of tumour growth, autophagy acts as a tumour suppressor, however, in the later stages of cancer, it undertakes a tumour promoter role [105]. However, a full understanding of the molecular mechanisms allowing autophagy to play a dual role in cancer is lacking. Only recently, autophagy was found to be involved in the survival of dormant BC cells and breast cancer stem cells (BCSC) [35,37,41]. Indeed, searching PubMed using the terms “autophagy”, “breast cancer” and “dormancy” only yielded 19 articles between 2008 and 2022. Furthermore, the induction of autophagy was shown to be one critical strategy used by dormant BCSCs for surviving a nutrient-deficient, hypoxic microenvironment that provides cells with energy through lysosomal recycling of cellular compartments, leading to dormancy [34,35,36,37,38,39,40,41]. For example, AKT and mTOR signalling pathways, fundamentally linked to autophagy, may be altered as a result of BC cells entering a dormant state after multiple cycles of hypoxia and reoxygenation [35]. Further to this, inhibiting autophagy can indeed impede the survival of dormant cells [37]. As such autophagy may be regarded as a crucial mechanism for the maintenance and survival of dormant BC cells. Furthermore, DTCs can remain dormant in the bone marrow, by upregulating autophagy which, for example, facilitated resistance to TRAIL cytotoxicity in the bone marrow [106], suggesting that in addition to survival, drug resistance in dormant cells may be impacted by autophagy. Therefore, in our opinion, there is currently an interest in targeting autophagy therapeutically to reduce the recurrence of dormant BCs. Hence, here, we have selected topics linking autophagy and dormancy with cell death, metabolism and the ECM and finally discuss treatment options.

### 3.3. Autophagy, Apoptosis, and Dormancy

Some of the first studies revealing a direct role of autophagy in dormancy were in the ovarian cancer field [107,108,109]. A study showed that the re-expression of the tumour suppressor Aplasia Ras homolog member I (*ARHI*, also known as *DIRAS3*) induced autophagy through inhibition of the PI3K/AKT/mTOR pathway and enabled cells to remain dormant in mice xenografts [81]. In BC, *ARHI* re-expression also enhanced the inhibitory effect of paclitaxel by promoting autophagic cell death (termed programmed cell death type II) and apoptosis [80]. These studies elegantly linked autophagy and dormancy with cell death.

Furthermore, Ramirez and colleagues recently showed that inhibiting the autophagic flux in dormant DTCs resulted in significantly reduced cell survival and metastatic burden both in vitro and in vivo [37]. Specifically, autophagy-related gene 7 (ATG7) knockout, inhibited mitophagy alongside autophagy, leading to an accumulation of damaged mitochondria and reactive oxygen species (ROS), resulting in apoptotic cell death. Interestingly, suppressing autophagy in proliferating cells did not induce apoptosis suggesting that autophagy is a critical survival process in dormancy that is deactivated after cells go through the dormant-to-proliferative switch [37]. In agreement with this study, Ovadia and colleagues found a link between dormancy and autophagy in a well-defined 3D ER+ dormancy model using bioinformatics analyses and experimental confirmation at the gene and protein level in BC T47D cells. Their work showed that ATG9B and LC3B were expressed at significantly higher levels in dormant cells than in proliferating cells [82]. In summary, in our opinion, autophagy may be used by dormant cells to survive, a process that is deactivated when these cells undergo a proliferative switch.

### 3.4. Autophagy, Metabolism, Nutrition, Hypoxia, and Dormancy

**Figure 4:**
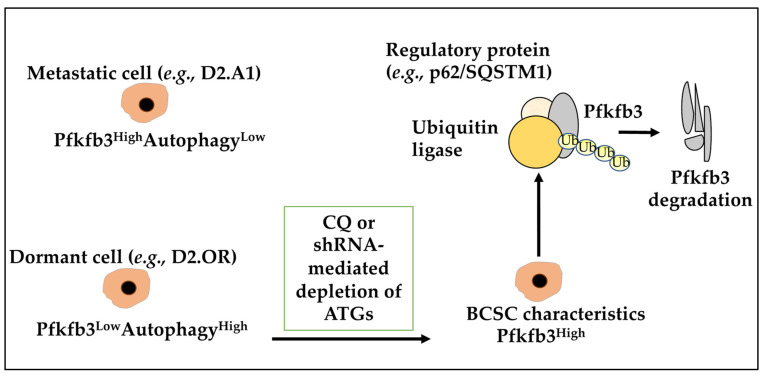
Metabolic characteristics of dormant cells. Dormant D2.OR BC cells displayed a low metabolism/high autophagy phenotype (for example, Pfkfb3^Low^Autophagy^High^), while their metastatic D2.A1 counterparts displayed a high metabolism/low autophagy profile (for example, Pfkfb3^High^Autophagy^Low^). Inhibiting autophagy in dormant D2.OR cells, either with chloroquine (CQ) or shRNA-mediated depletion of ATGs, induced expression of Pfkfb3 and BCSC characteristics. Pfkfb3 is an autophagy substrate that binds to a regulatory protein (for example, p62/SQSTM1) contributing to dormancy partly by enabling p62/SQSTM1-mediated degradation of Pfkfb3.

Many metabolic processes may be linked to autophagy in dormant cells. The critical glycolysis mediator 6-phosphofructo-2-kinase/fructose-2,6-biphosphatase 3 (Pfkfb3) was investigated and showed its expression to correlate inversely with autophagy levels where dormant D2.OR cells display a Pfkfb3^Low^Autophagy^High^ phenotype, while their metastatic D2.A1 counterparts display a Pfkfb3^High^Autophagy^Low^ phenotype [83,110]. Indeed, inhibiting autophagy in D2.OR cells, either with chloroquine (CQ) or shRNA-mediated depletion of ATGs, induced BCSC expression of Pfkfb3 and emergence of metastatic dormancy [83]. Moreover, the study found that Pfkfb3 is an autophagy substrate that binds to p62/SQSTM1 (a ubiquitin-binding scaffold protein), highlighting that autophagy activation contributed to metastatic dormancy partly by enabling p62/SQSTM1-mediated degradation of Pfkfb3, underlining metabolic properties of dormant cells (Figure 4) [83]. Moreover, hypoxia is also known to induce tumour cell dormancy, and de Prati and colleagues have found that under chronic hypoxia, metastatic BC cells enter into a dormant state where AMP-activated protein kinase (AMPK) activation and increased autophagy levels sustained their survival [35]. In our opinion, the increase in metabolic outputs and hypoxia-induced autophagy can impact dormancy and dormant cell awakening.

In addition, the link between autophagy, calorie restrictions, and DNA damage in BC has also been investigated. In evidence, calorie restriction is also known as “fasting” and can lead to the induction of DNA repair and autophagic mechanisms and heat-shock chaperones. Consistently, cell proliferation, ROS, ageing and cell senescence were reduced, while anti-tumour immunity was augmented as a result of calorie restrictions [111,112]. Further, it has been established that oestrogen, insulin growth factor 1 (IGF-1) and testosterone lead to elevated levels of DNA damage and equally defective DNA damage response mechanisms [113], while calorie restrictions reduced growth factors, anabolic hormones and inflammatory factors [114]. The anti-cancer effect of calorie restrictions may therefore be linked to decreased systemic levels of IGF-1. To investigate this link in greater detail, a study compared the effect of calorie restrictions in the presence and absence of exogenous IGF-1 on immunodeficient mice xenografted with mammary tumours. This study identified that genes and pathways linked to steroid hormone metabolism and also nutrients were correlated with the anti-tumour effect of calorie restrictions. Inversely, exogenous IGF-1, as expected, rescued several genes and pathways linked to tumourigenesis and metabolism that were affected by calorie restrictions. Hence, reduced IGF-1 could account for some of the beneficial effects of calorie restrictions, while increased IGF-1 could reverse the tumour inhibitory effects of calorie restrictions [115].

### 3.5. Autophagy, ECM, and Dormancy

For tumour cells to successfully metastasise, they must survive conditions without a stable ECM contact, where integrin and growth factor signalling is compromised. In normal cells, an aberrant ECM attachment triggers anoikis (programmed cell death), however, tumour cells employ mechanisms that resist anoikis as well as autophagy [64,84,85,86]. Failure of DTCs to engage with a new ECM has been shown to trigger tumour dormancy Specifically, inhibiting β1 integrin signalling promoted dormancy by elevating autophagy in the mouse mammary tumour virus-polyoma middle tumour-antigen (MMTV-PyMT), a mouse mammary gland carcinoma model of BC [64,84,85,86]. This autophagy upregulation allowed BC cells to establish cell-ECM contacts necessary to survive at secondary sites (Figure 2). Moreover, an increased autophagic flux, maintained BC dormancy via downmodulation of E-cadherin, a major component in cell-cell adhesion and epithelial tissue homeostasis and whose downregulation is linked to tumour progression (Figure 2) [64,84,85,86]. Therefore, autophagy upregulation in ECM-detached cells may compensate for the loss of extrinsic signals, thereby promoting nutrient and energy metabolism and maintenance of the dormant state [64,84,85,86].

The biomechanics of the host tissue may also impact dormancy. For instance, BC relapse can be frequently identified in tissues that are softer than the primary breast tumour, such as bone marrow and lung [87]. The authors found that in soft microenvironments, BC cells were more resistant to tamoxifen as a consequence of increased autophagic activity (an increase in the expression of LC3B-II and a decrease in p62 levels were seen in MCF-7 (ERα^+^ BC cells) or ZR-75-1 cell cultured on soft substrata). Indeed, using siRNAs to reduce the levels of Atg7 or Beclin-1, MCF-7 cells became sensitised to tamoxifen treatment. Interestingly, they found that autophagy levels decline as substratum stiffness increases [87]. In conclusion, dormant cells could persist in the soft microenvironment due to autophagy.

**Figure 5:**
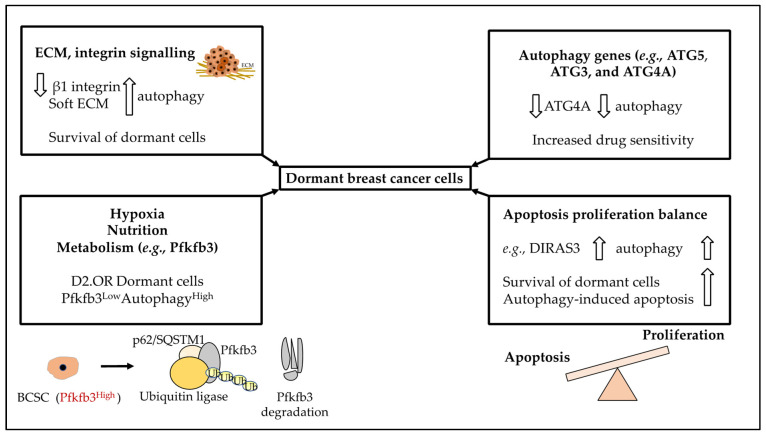
The summary of the contribution of autophagic processes to dormant cells. Autophagy, through its various associated genes and players including but not limited to ATG5, ATG3 and ATG4A can impact dormancy. For example, the downregulation of ATG4A can lead to reduced autophagy and increased drug sensitivity in BC cells (and reduced dormancy). Furthermore, autophagy can impact the balance of apoptosis and proliferation, for example, DIRAS3 upregulation could increase autophagy, dormant cell survival and also cell death due to autophagy. Moreover, autophagy is linked to metabolism, nutrition and hypoxia and through this can impact dormant cells, for instance, dormant BC cells could downregulate *Pfkfb3*, a gene involved in metabolism. As discussed earlier, inhibiting autophagy induced BCSC expression of Pfkfb3 and the emergence of metastatic dormancy, while Pfkfb3 protein could be degraded by p62 and ubiquitin ligase complexes. Finally, loss of contact with other cells and the basement membrane (for example through integrin and E-cadherin downregulation) in addition to a soft ECM can increase autophagy levels and enhance dormancy.

In our opinion, the ECM, integrin and growth factor signalling in addition to the stiffness of the secondary tissue are strongly linked to autophagy and this can impact dormancy. In Figure 5 we have summarised the role of autophagy in BC dormancy.

### 3.6. Inhibiting Autophagy as a Therapeutic Strategy in BC

Autophagy may play disparate roles during different stages of tumour development and treatment response. In that respect, autophagy is regarded as a double-edged sword in that it can suppress tumours by removing damaged proteins and organelles and limiting growth, as opposed to conferring stress tolerance in tumourigenesis [116]. Here, we have examined both scenarios.

In evidence, the sensitivity of BC cells to hydroxychloroquine (HCQ), an autophagy inhibitor through blocking autophagosome/lysosome fusion, was assayed using proliferation and invasion assays, while the activation of autophagic and signalling pathways was also monitored. Notably, SUM-190 cells representing basal-like BC subtype, showed a relatively higher sensitivity of HCQ compared to other BC cell lines, whereby both proliferation and migration were significantly reduced, while levels of LC3-I/II, p62 (SQSTM1) and Ras/Raf/ERK signalling were also downregulated [117]. In another study, the delivery of docetaxel with siRNA targeting the ATG7 was proposed by Gong and colleagues. ATG7 is involved in the formation of autolysosomes and the clearance of damaged mitochondria, while downregulation of ATG7, led to increased levels of caspase 3/9-mediated apoptosis [118]. The combination treatment of ATG7 siRNA and doxorubicin significantly enhanced apoptosis rates and cytotoxicity in MCF-7 BC cell lines. The effective delivery of the ATG7 siRNA and doxorubicin was achieved by utilising polypeptide micellar systems comprising lipoic acid and a cytosol localisation and internalisation peptide, allowing for a significant accumulation of these micelles in the tumour cells. This combination reduced autophagy-mediated doxorubicin resistance and increased the apoptotic and anti-tumour effects of this chemotherapeutic agent [118]. These two studies, therefore, drive the premise that autophagy blockage can reduce BC cell proliferation and enhance treatment efficacy.

As discussed earlier, autophagy is a double-edged sword in tumourigenic processes. For example, Li and colleagues have shown that autophagy-deficiency led to TNBC progression by limiting T-lymphocyte mediated immunity, while this immunosuppression may have been mediated by Tenascin-C (TNC) an ECM glycoprotein involved in migration, proliferation and angiogenesis [119,120]. Specifically, this study using in vitro models showed that blocking autophagy led to decreased cytotoxic T lymphocyte-mediated immunity, while the trafficking of tumour-reacting T cells to the tumour cells was reduced in vivo. These autophagy-deficient tumours, as expected, did not respond well to anti-PDL1 treatment. This study showed that autophagy deficiency in TNBC may explain checkpoint inhibitor resistance in this subtype, a result that is in contrast to the 2 other studies discussed in this section [120].

### 3.7. Autophagy, Dormancy and BC Treatment

Further to reviewing the role of autophagy in BC treatment, we will now focus on this role from the angle of BC tumour dormancy and how autophagy-based treatment options may impact dormancy in BC. We reviewed the role of DIRAS3, which upon expression in BC leads to reduced PI3K/AKT signalling, angiogenesis and cancer growth. In addition, DIRAS3 triggered autophagy both in vitro and in mice xenograft models and facilitated the survival of dormant cells; hence, targeting this gene may eliminate these dormant cells as a therapeutic strategy [88]. In addition, Aqbi and colleagues investigated the role of autophagy in relapse occurrence in HER2 (neu) expressing mouse mammary carcinoma (MMC) model receiving Adriamycin. In this study, the stable knockdown of ATG5 led to the permanent blockage of cell-intrinsic autophagy, a type of autophagy that protected cells from toxin-induced genomic instability. Downregulated ATG5 led to reduced sensitivity to the chemotherapy treatment and early dormancy escape [89]. Hence, these two studies aligned since the expression of a protein triggering autophagy led to dormant cell survival, and in the latter study, the downregulation of an autophagic gene led to reduced dormancy. Despite this, in our opinion, the role of autophagy in inducing or delaying dormancy must be understood on a context-dependent level.

In this section, we have addressed the link between autophagy and dormancy from numerous perspectives including apoptosis, nutrition, metabolism, hypoxia, and ECM. The induction of autophagy has shown to be an important pro-survival strategy used by BCSCs and dormant BC cells and as such many efforts are currently taking place in targeting autophagy therapeutically to minimise metastatic recurrence. However, whether keeping cells dormant or forcing their “awakening” is still controversial; for example, “autophagy addiction” in chemotherapy-resistant cells, could be targeted to eliminate disseminated dormant cells in patients [121]. On the other hand, as highlighted above in the dormant-to-proliferative switch, blocking autophagy might reactivate cells to proliferate again.

We also highlighted that a selective form of autophagy, mitophagy, is activated in the dormant-to-proliferative switch study. However, the role of selective autophagy in cancer dormancy remained understudied, with only a few studies available. Indeed, the development of effective autophagy-modulating drugs has been hindered by the paradoxical role of autophagy in tumourigenesis. Therefore, future studies could address to which degree chemotherapy-induced cytotoxicity indeed depends on autophagy modulation. Moreover, developing biomarkers that can identify patients most likely to benefit from autophagy modulation could be considered [34].

## 4. The Role of lncRNAs, Exosomes and miRNAs in BC Dormancy

Long non-coding RNA (lncRNAs) are transcripts longer than 200 base pairs expressed specifically at low levels in cells and tissues and are responsible for cell and tissue-specific activities. LncRNAs are involved in physiology and disease and have gained much attention as potential therapeutic targets and cancer biomarkers and may play significant roles in the processes of tumourigenesis and tumour suppression [43]. LncRNAs can regulate various cancer processes including metastasis, proliferation and survival [122]. In BC, *MALAT1* contributed to tumour proliferation, progression and metastasis in TNBC [123], while *HOTTAIR* has been proposed as a predictor of metastatic progression in BC [124]. Moreover, in BC, lncRNAs *NEAT1* and *PTENP1* have been shown to play roles in tumour progression in BC [125,126]. In addition, miRNAs have been linked to numerous tumourigenic processes in BC [127], these are single-stranded conserved non-coding RNA, that target mRNAs through the RNA-induced silencing complex (RISC) [128].

On these grounds, it is possible to postulate that specific lncRNAs and miRNAs may be linked to BC recurrence, the evidence for which we have presented in this section.

### 4.1. The Role of lncRNAs and miRNAs in BC Dormancy

**Figure 6:**
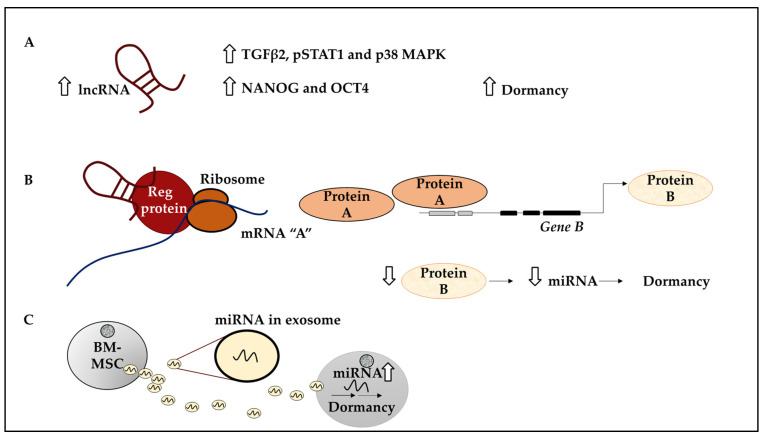
Underlying mechanisms of action of lncRNAs in inducing dormancy in BC. (**A**) Increased levels of lncRNAs (for example, *NF2R1-AS1*) can lead to increased levels of dormancy mediators including TGFβ2, pSTAT1 and p38 MAPK, while stemness markers such as OCT4 and NANOG may also be increased. (**B**) LncRNAs (for example, *NF2R1-AS1*) can promote metastatic dormancy by binding to a regulatory protein (for example, PTBP1) which can increase the translation of *mRNA A* and the production of protein A. Protein A (for example, NR2F1) per se can reduce the transcription of *gene B* (for example, *TP63*). Reduced levels of protein B (for example, ΔNp63) can lead to the modulation of regulatory miRNAs (for example, *miR205*) influencing dormancy. (**C**) Exosomes produced by BM-MSCs containing miRNA that influence BC cell dormancy.

A strong link has been established between lncRNA *NR2F1-AS1* and tumour recurrence. A study by Sanchez Calle and colleagues has revealed the relationship between *NR2F1-AS1* and ER+ BC cells and ER+ primary tissue [67]. This study reported an inverse correlation between *NR2F1-AS1* and PR, and a weak correlation between ER and *NR2F1-AS1* [67]. A more detailed analysis revealed that the elevated expression of *NR2F1-AS1* was correlated with poor OS and distant metastasis-free survival (DMFS) [67]. *NR2F1-AS1* expression was also tested in 9 BC cell lines and higher expression of this lncRNA was found in TNBC and HER2+ subtypes and the ER+ luminal A subtype inclusive of MCF-7 and T47D [67]. In addition, ChIP-qPCR analysis in MCF-7 and T47D revealed the enrichment of PR over ER at the *NR2F1-AS1* promoter regions in T47D compared to MCF-7 cells. This suggested that ER and PR may play a role in transcriptional regulation of *NR2F1-AS1* which was confirmed by increased levels of *NR2F1-AS1* following the depletion of PR and ER. Furthermore, at least 2 variants of *NR2F1-AS1* including Var1 and Var4 were identified. The overexpression of either *NR2F1-AS1* variants by stable transfection in the BT474 cell line led to the induction of a quiescence-like state in ER+ BC cells marked by decreased Ki-67 levels [67]. Further, markers of dormancy including TGFβ2, pSTAT1 and p38 MAPK, and markers of pluripotency such as NANOG and OCT4 were overexpressed in BT474-Var1 cells (Figure 6A) [67]. In summary, this study revealed that a specific lncRNA, *NR2F1-AS1* through its Var1 and Var4 variants could influence BC dormancy, a useful link for future studies aiming to eliminate these cells in BC.

Further, many studies have drawn comparisons between quiescent dormant DTCs and cancer stem cells (CSCs) in that dormant DTCs may display stem-like characteristics, as mentioned earlier [25,129]. BCSCs may express CD44^high^ CD24^low^ [130]. In a study by Liu et al., the role of *NR2F1-AS1* was further investigated in BCSCs medium and high expressing subpopulations; CD44^med^ CD24^−^ (P1) and CD44^high^ CD24^−^ (P2) and their representative cell lines. Accordingly, the MCF10CA1h cell line contained both P1 and P2, while MCF10CA1a containing P1 cells were more metastatic and expressed aldehyde dehydrogenase 1, ALDH1^+^ [23]. To study the post-dissemination metastatic potential of these cells, MCF10CA1a and MCF10CA1h were labelled with luciferase and GFP and injected into athymic mice intravenously [23]. This revealed that a higher number of GFP-expressing MCF10CA1h were seeded in the lungs compared to MCF10CA1a [23]. Also, MCF10CA1a cells developed lung metastasis, while MCF10CA1h cells remained as single cells or small foci, suggesting that they were not metastatic [23]. Notably, *NR2F1-AS1* was expressed in MCF10CA1h compared to metastatic nodules and the knockdown of *NR2F1-AS1* led to fewer injected GFP-positive cells seeding in the lungs. Consistently, *NR2F1-AS1* overexpression in MCF10CA1a reduced tumourigenic capacity [23]. This data suggested that *NR2F1-AS1* promoted the seeding of tumour cells and inhibited their proliferation, leading to the dormancy of BC cells in the lungs. The underlying mechanism was revealed as *NR2F1-AS1* binding to *NR2F1*, thereby recruiting the RNA binding protein, PTBP1, to promote NR2F1 translation, leading to reduced ΔNp63 (*TP63* gene variant) transcription mediated by NR2F1. ΔNp63 downregulation led to increased metastatic dormancy of BC cells in the lungs via regulating *miR-205* [23]. This study provided a direct mechanistic link between lncRNA and miRNA function and the induction of dormancy, which may be exploited to design targeted therapy (Figure 6B). This study also highlighted similarities between BCSCs and dormant cells concerning surface markers and in vivo function. For example, CD44^high^ CD24^−^ ALDH1^−^ cells were more quiescent than their ALDH1^+^ counterparts.

In a more recent study, ESR1 locus enhancing and activating non-coding RNAs (*ELEANORS*) was investigated in ER+ BC. The emergence of recurrence in this subtype is high, hence it stood to reason to investigate potential lncRNAs that may contribute to dormancy in this subtype [131,132]. This lncRNA pertained to the ESR1 locus and belonged to a cluster of lncRNAs identified in recurrent BC. The function of these lncRNAs, however, has been identified as chromatin regulators in various in vitro models [131,132], but the current study investigated their link with clinicopathological attributes of BC patients using primary and matched metastatic BC models [132]. Accordingly, this study identified that *ELEANORS* expression was specific to ER+ BC, while its expression correlated with ER and PR expression in both models [132]. Interestingly, *ELEANORS* was expressed in both primary and metastatic tumours. Notably, the incidence of relapse was greater if the patient displayed *ELEANORS* expression after 5 years post-surgery. The use of Cox regression to investigate uni- and multi-variate regression concluded the expression of *ELEANORS* were independent risk factors of disease recurrence [132]. This study also shed light on the underlying mechanism of action of *ELEANORS* in cell lines, patient primary tissue and mouse xenograft models and showed that *ELEANORS* upregulated BCSCs-related genes including CD44, and through the maintenance of stemness characteristics influenced BC dormancy [132].

### 4.2. The Role of lncRNAs and miRNAs in BC Dormant Cell Awakening

In addition to reporting on the roles of lncRNAs in inducing dormancy in BC, recent evidence has shed light on the role of lncRNAs in the awakening of dormant cells. LncRNA *BORG* may contribute to the ability of disseminated BC cells to survive and alter their proliferation profile. Specifically, *BORG* expression was upregulated in ER- BC over their ER+ indolent counterparts, while TNBC samples expressed higher levels of *BORG* compared to normal mammary tissue [90]. Consistently, highly metastatic D2.A1 expressed higher levels of *BORG* and this endowed D2.OR cells with a higher proliferative advantage. Low expression of *BORG* correlated with an indolent non-proliferative state in BC [90]. Furthermore, *BORG*-expressing D2.OR cells injected into the BALB/c mouse model, displayed efficient colonisation potential compared to the parental D2.OR which completely lacked this potential [90]. Immunohistochemical analysis of the *BORG*-expressing D2.OR cells displayed increased Ki-67 marker expression by these cells [90]. Contrastingly, *BORG*-depleted D2.A1 cells injected into BALB/c mice displayed significantly reduced lung metastasis, collectively lending support to the notion that *BORG* induced metastatic properties [90]. The study revealed the underlying mechanism of *BORG* action as promoting the localisation and transcriptional activity of TRIM28 and E3 SUMO ligase. TRIM28 in turn functioned to suppress the expression of *p21* and *gadd45a*, leading to BC proliferation, while the TRIM28/*BORG* axes drove the reactivation of latent disseminated BC cells [90] (Figure 6C). This section, hence, highlighted the importance of two lncRNAs (*NR2F1-AS1* and *BORG*) and their associated miRNAs in influencing BC dormancy, which may be significant targets for therapeutic gain in BC.

In addition, the role of metastasis-associated lung adenocarcinoma transcript 1 (*MALAT1*) in BC dormancy was investigated [91]. In this study, *MALAT1* was revealed as a lncRNA involved in the reactivation of dormant BC cells. Using a myriad of in vitro and in vivo models and tools such as single-cell RNA-seq, ChIP-seq and flow cytometry, the authors showed that the deletion of this lncRNA completely abolished the metastasis potential of BC cells, while the overexpression of *MALAT1* led to the awakening or reactivation of these dormant BC cells [91]. Consistently, the deletion of *MALAT1* dampened cancer stemness characteristics and immune evasion. Interestingly, the injection of the *MALAT1* knockout BC cells into the mouse model devoid of sufficient T-cell immunity led to the inhibition of metastatic outbreak of these cells. The underlying mechanism was explained through the network of *MALAT1*- *Serpinb6b*, whereby expressing *Serpinb6b* in *MALAT1* knockout cells led to a metastatic outbreak, since *Serpinb6b* could protect cells against T cells and promote metastasis. In our opinion, the role of *MALAT1* in enabling metastatic outbreak could be a useful link to targeting this lncRNA and preventing the awakening of dormant BC cells [91]. Further to the role of lncRNAs in the awakening of dormant BC cells, evidence suggests that circulating miRNAs may predict the recurrence of BC in patients. In evidence, a study investigated the significance of various miRNAs including *miR-200c* in predicting disease recurrence in patients diagnosed with early stages of BC. Accordingly, the higher expression of *miR-200c* correlated with reduced disease-free survival, while this miRNA was highly expressed in both relapse and late relapse patient samples [133]. In our opinion, the evidence presented in this section has shed light on the significance of lncRNAs and miRNAs in dormant cell awakening, suggesting that these non-coding RNAs may be used as predictive biomarkers or targets for treatment.

### 4.3. The Role of Exosomes in BC Dormancy

Exosome-enclosed miRNAs may also be linked to dormancy. Exosomes are intraluminal vesicles formed by exocytic fusion with the cell membrane that may contain intracellular components including mRNAs, protein and miRNAs and display an average size of 100 nanometres in diameter [134]. These vesicles produced by cancer cells may impact many processes such as angiogenesis and immune cell infiltration [44,45]. In turn, the cells of the microenvironment can also produce exosomes that can influence cancer cells [135]. In a study aimed at understanding the impact of the exosomes generated by the BM-MSCs on BC cells, labelled MDA-MB-231 cells injected to C.B-17/Icr-scid/scidJc1 mice were found to disseminate and home in the bone marrow and were termed BM2 [46]. BM2 cells were revealed to express low CD24 surface markers, akin to CSCs [46]. Furthermore, BM2 cells were co-cultured with BM-MSCs characterised by expressing CD73, CD90 and CD105 and were also labelled with PKH26 dye retained by slow-cycling or dormant cells [46]. The co-culture of BM2 cells with human BM-MSCs confirmed a shift in BM2 cells towards dormancy, whereby a trend was observed for decreased numbers of BM2 cells in G2/M and an increase in G0/G1 phases [46]. This study also revealed that when PKH26-labeled BM2 cells were treated with secreted exosomes from BM-MSCs, higher levels of PKH26 dye abundance were obtained, suggesting increased levels of dormancy induced in BM2 cells. BM2 cells cultured for up to a week in the presence or absence of BM-MSC-derived exosomes were also orthotopically transplanted to the mammary fat pads of SCID mice [46], resulting in reduced cell proliferation in BM2 cells treated with BM-MSC-derived exosomes. Interestingly, the exosomes generated by BM-MSCs were shown to be enriched for *miRNA-23b*, a miRNA suppressing *MARCKS*, a gene involved in the cell cycle, whereby *miR-23b* from BM-MSCs decreased *MARCKS* expression in BM2 cells, resulting in dormancy [46]. In our opinion, this study demonstrated the effect of resident stem cells in the tumour microenvironment which influenced dormancy in the neighbouring tumour cells.

A similar study showed that BC cells primed BM-MSCs to produce *miR-222/223* containing exosomes which *per se* promoted dormancy in BC cells [92]. While Lim and colleagues also reported that miRNA-containing exosomes including *miR-222/223*, *mir-127* and *mir-197* produced by the BM stroma induced dormancy in BC cells, as did the transmission of these miRNA through gap junctions [93]. Interestingly, the small size of the miRNA makes them an excellent candidate for transfer through these gap junctions. Accordingly, this passage allowed for the facilitation of gap junctional intercellular communication and the flow of information between BC cells and the BM-MSCs and the subsequent dormancy induction in BC cells [136]. The authors showed that co-culturing of BC cell lines inclusive of T47D with the bone marrow stroma containing miRNA-enclosed exosomes induced cell cycle arrest and quiescence in BC cells, through the suppression of CXCL12, a gene involved in proliferation [93]. In addition, BC cells primed with MSC exosomes were more highly resistant to chemotherapy [137].

Finally, the role of exosomes generated by MSC affecting MDA-MB-231 cells was also investigated. The authors revealed that exosome-enclosed miRNA including *miR-205* and *miR-31* induced dormancy in MDA-MB-231 cells, whereby the ubiquitin-conjugating enzyme E2N (UBE2N/Ubc13) was a target of these miRNAs and the downregulation of this enzyme led to the suppression of invasion and proliferation [94]. In our opinion, the modulation of the miRNAs mentioned in this section, including *miR-23b*, may allow for the BC cells to remain dormant and prevent their potential awakening.

### 4.4. DNA Repair, Exosomes, and BC Dormancy

There may be links between the induction of dormancy, DNA damage repair response and exosomes. In a study, the dynamics of the BC disseminated cells which enter the BM perivascular niche were studied in greater detail. For instance, one of the first processes was the acquisition of dormancy and stemness characteristics including DNA repair capacity and cell cycle quiescence, which collectively could be induced by MSCs and their respective exosomes [138]. In addition, the MSCs also protected the dormant BC cells from immunosurveillance by skewing the TME towards immune tolerance [139]. This study utilised single-cell RNAs-seq to uncover the various pathways that may be activated in response to BC cell exposure to exosomes. In this study, naïve and primed exosomes were collected from MSCs which had never been in contact with BC cells and MSCs which had been in contact with BC cells, respectively. Subsequently, the genes activated in BC cells following exposure to naïve and primed exosomes were then determined [138]. For example, in the former, genes involved in mismatch repair, nucleotide excision repair and DNA double-strand breakage were observed, while for the latter genes relating to the Wnt/β-catenin pathway, necessary for the completion of dedifferentiation, were enriched. The authors hypothesised that DNA repair (including but not limited to mechanisms such as basic excision repair (BER) and ten-eleven translocation (TET)) and Wnt signalling pathways may indeed be required for the completion of dedifferentiation of BC cells to cells with stemness and dormant characteristics, and this was accomplished by two waves of exosomes generated by MSCs [138]. In our opinion, this study highlighted the significance of stromal cells in inducing dormancy by activating pathways necessary for change.

Interestingly, the flip side of the coin is also possible in that BC cells can induce cells within the tumour microenvironment and, through that, impact DNA damage-related processes. In evidence, in a study 3 BC cell lines were investigated including MCF7, MDA-MDB-231, and T47DA18 and the exosomes generated by these cells to signal primary mammary epithelial cells were studied [140]. Interestingly, the exosomes generated by the BC cells induced DNA damage and autophagy and generated ROS. Specifically, genes including *ATM*, *H2AX*, and *CHK1* were activated, indicating that DNA damage repair processes had indeed been triggered, while p53 was also activated. Notably, autophagy, DNA damage responses, and p53 activation could be mitigated by N-acetyl-L-cysteine, effectively linking DNA damage to autophagy and p53 activation. The authors hypothesised that BC cells manipulated the tumour microenvironment through the mentioned mechanism to facilitate their growth and survival [140]. In our opinion, these pathways can ultimately affect the induction of dormancy. The evidence reviewed in this section highlighted the role exosomes played in priming dormant BC cells in addition, BC cells could induce alterations in their microenvironment, and both processes involved DNA damage repair mechanisms.

## 5. Discussion; Aspects of Autophagic Processes, lncRNAs and miRNAs for Targeted Therapy in BC

Further to discussing the roles of autophagy, lncRNAs and miRNAs in this section, we aim to critique the potential utility of these links for therapy. In the field of BC, the mTOR inhibitor, everolimus, has been used as an antitumour treatment option in this cancer by impeding autophagy and inducing dormancy, which highlights very significant aspects of BC therapy; keeping the cells dormant [141]. On the other hand, we know that autophagy is closely linked to apoptosis; both the induction and inhibition of autophagy have been reported to promote tumour cell apoptosis [142,143]. The combination of chemotherapy and radiotherapy sensitisers; pentoxifylline (PTX) and simvastatin (SIM) drove dormant autophagic MDA-MB-231 BC cells to undergo apoptosis, therefore highlighting a novel treatment strategy for clinical cancer resistance [143]. In addition, liensinine may block the autophagosome-lysosome fusion stage of autophagy, by inhibiting the recruitment of RAB7A, while the inhibition of autophagy using liensinine with chemotherapeutic agents enhanced apoptosis in BC cells [144]. These studies suggested that autophagy-induced apoptosis can be exploited for therapeutic gain once the linked mechanism is understood.

Furthermore, we were also interested in understanding how autophagic processes, therapy and dormancy might be linked. For example, a combination treatment of small molecule inhibitors and autophagy inhibitors may be useful in targeting dormant cells. For instance, human protein farnesyltransferase inhibitors (FTIs), which have been successful at suppressing tumour growth in pre-clinical studies [145], have been shown to induce dormancy in MCF-7 cells by upregulating protein expression levels of autophagy markers such as the Atg12 and Atg5 conjugate [146]. The authors found that induction of autophagy allowed the cells to maintain the FTI-induced dormant phenotype, and indeed co-treatment of FTI and the autophagy blocker 3-MA led to a significant reduction in cells presenting a dormant phenotype [146]. Hence, it is plausible to suggest that combination therapy with autophagy inhibitors could reduce the burden of dormant cells and therefore reduce the possibility of disease recurrence, a useful link to take forward.

Other combination therapies may also be useful; combining proteasomal and autophagy inhibitors with chemotherapeutic compounds might be one way of tackling the autophagy-induced chemotherapy resistance and dormancy [34]. For instance, the novel proteasome inhibitor, MLN9708, enhanced the sensitivity of BC cells to doxorubicin-induced apoptosis in a manner that was inversely related to autophagy levels [147]. The study agreed with other experiments that highlighted the significance of autophagy in the resistance to proteasome inhibitor treatment (for example, MG132) in BC cells [148]. This study is very elegantly linked to tackling autophagy-mediated drug resistance by using a different compound, including a proteasomal inhibitor, which may interfere with autophagy-fuelled protein degradation processes and impede autophagy.

Concerning lncRNAs, miRNAs and exosomes, we reviewed gene regulatory networks which may be targeted for therapy. For instance, *NR2F1-AS1* in BC was linked to increased levels of stemness and dormancy markers and through mechanisms discussed led to BC dormancy [23,67]. Therapeutic interventions may therefore include modulating *NR2F1-AS1* to reduce dormancy and perhaps sensitise the cells to therapy. In contrast, we reviewed *BORG* that in cooperation with TRIM28 facilitated the reactivation of latent dissemination BC cells [90]. Hence, preventing the awakening of dormant cells by potentially modulating lncRNAs promoting dormant cell reactivation, including *BORG* or *MALAT1*, could be a plausible route forward [90,91]. These lncRNAs may be targeted using several methods for lncRNA inhibition including locked nucleic acid, morpholinos, antisense nucleotides and single-stranded DNA polymers [149,150,151]. Finally, we reviewed miRNAs encapsulated by exosomes produced by BM-MSCs [46,92], which may be targeted by miRNA inhibitors to reduce dormancy induction and eradication of dormant cells by therapy. Exosomes also primed BC cells to become dormant and thereby activated DNA repair mechanisms [138], while it was also conceivable that BC cells could prime cells in the TME to promote their growth and survival [140].

In conclusion, despite advances in cancer therapies, a fraction of BC patients displaying no evidence of systematic dissemination at primary cancer diagnosis and treatment can develop recurrent disease over time. This may be due to the early dissemination of BC cells that have not been detected by conventional methods. Hence, the therapeutic targeting of disseminated BC is of high importance. These cells may also be resistant to conventional chemotherapy targeting rapid cycling cells, highlighting the significance of understanding the underlying mechanism of subversion and regrowth. Therapeutic strategies discussed may involve autophagy and lncRNA-miRNA axes and enable the prevention of dormancy, the depletion of dormant residual tumour cells post-therapy, and their sensitisation to therapy or the prevention of the awakening of dormant cells.

## Figures and Tables

**Table 1:** Examples of factors influencing the induction of dormancy or awakening of BC cells.

Factors Influencing the Induction of Dormancy	Example of Effect and Mechanism Involved	Source
p38 MAPK/ERK TGFβ2 (and TGFβ-RIII)	TGFβ2 (and TGFβ-RIII) and EDG2 can lead to an enhanced ratio of the p38 MAPK/ERK levels and dormancy	[24,48,49]
Immunosurveillance	Loss of MHC class I or loss of tumour antigens leading to tumour cell survival	[53]
Angiogenic dormancy	Downregulation of pro-angiogenesis factors and the production of suppressors of angiogenesis impacting dormancy	[56,57]
FGF-2	Growth and proliferation inhibition by inducing p21, leading to G1 cyclin complex inactivation in BC	[62,63]
α5β1 integrin-fibronectin	Survival of FGF2-responsive BC cell in the bone marrow	[64]
LIFR/STAT3/SOCS3	The loss of STAT3 and LIFR in BC cells reduced dormancy and CSC markers and promoted proliferation	[65]
mTORC1/mTORC2, TGFβ2, BMP, NR2F1 and DEC2 (BHLHB3)	Induced dormancy	[48,66,67,68]
*DNMT1* and *CDKN1A*	5-Azacytidine induced p38-induced dormancy signature (e.g., reduced *DNMT1*, and increased *CDKN1A*)	[70,73,74]
Tumour microenvironment stromal cells	BC cell interaction with E-selectin+ ECs leading to maintenance of dormant state	[51,57,69]
Chronic inflammation, smoking, remodelling of the extracellular matrix, and signalling cascades converging on ERK	Promoted growth and awakening of dormant cells	[75,76,77,78,79]
*ARHI* (*DIRAS3*) re-expression	Induced autophagy by blocking PI3K/AKT/mTOR and enabled cells to remain dormant	[80,81]
Autophagy-related gene 7 (ATG7) knockout	Inhibited mitophagy alongside autophagy, leading to an accumulation of damaged mitochondria and reactive oxygen species, resulting in apoptotic cell death in dormant cells	[37]
ATG9B and LC3B	Expressed at significantly higher levels in dormant cells than in proliferating cells	[82]
Inhibiting autophagy in D2.OR cells, either with chloroquine (CQ) or shRNA-mediated depletion of ATGs	Induced BCSC expression of Pfkfb3 and emergence from metastatic dormancy	[83]
Inhibiting β1 integrin signalling	Prompted dormancy in the MMTV-PyMT model of BC, which can induce autophagy to give cancer cells time to establish cell-ECM contacts necessary to survive at secondary sites	[64,84,85,86]
Autophagy upregulation in ECM-detached cells or BC cells in soft substrata	Maintenance of the dormant state	[87]
DIRAS3	DIRAS3 triggered autophagy both in vitro and in mice xenograft models and facilitated the survival of dormant cells	[88]
ATG5	The in vitro and in vivo models of shRNA-mediated knockdown of ATG5, demonstrated reduced sensitivity to the chemotherapy treatment, inducing early escape from dormancy	[89]
Var1 *NR2F1-AS1* expressing BT474 cells	Upregulation of markers of dormancy including TGFβ2, pSTAT1 and p38 MAPK and markers of pluripotency such as NANOG and OCT4	[67]
*NR2F1-AS1*	*NR2F1-AS1* binding to NR2F1, leading to ΔNp63 transcription and increased dormancy of BC cells	[23]
*BORG*	Promoting transcriptional activity of TRIM28 leading to suppression of p21, TRIM28/*BORG* axes drove the reactivation of latent disseminated BC cells	[90]
*MALAT1- Serpinb6b axis*	Expressing *Serpinb6b* in *MALAT1* knockout cells led to metastatic outbreak, since *Serpinb6b* could protect cells against T cells	[91]
*miRNA-23b*	Exosome generated by BM-MSCs enriched for *miRNA-23b*, suppresses MARCKS (a cell cycle gene) resulting in dormancy	[46]
*miR-222/223*	BC cells primed BM-MSC to produce *miR-222/223* containing exosomes which *per se* increased dormancy in BC cells	[92]
Stroma generated miRNA-containing exosomes including *miR-222/223*, *mir-127* and *mir-197*	Induced dormancy in BC cells through suppressing CXCL2	[93]
Exosome-enclosed miRNAs including *miR-205* and *miR-31*	Induced dormancy in MDA-MB-231 cells by downregulating ubiquitin-conjugating enzyme E2N (UBE2N/Ubc13) and the resulting suppression of invasion and proliferation	[94]

## Data Availability

Not applicable.

## Abbreviations

ALDH	Aldehyde dehydrogenase
AMPK	AMP-activated protein kinase
ARH1	Aplasia Ras homolog member I
ATG	Autophagy-related
BER	Basic excision repair (BER)
BM	Bone marrow
BM-MSCs	Bone marrow mesenchymal stem cells
BC	Breast cancer
BCSC	Breast cancer stem cells
C.B-17/Icr-scid/scidJc1mice	Severe combined immunodeficient mice
CQ	Chloroquine
CSCs	Cancer stem cells
DMFS	Distant metastasis-free survival
DNMT1	DNA methyltransferase 1
DTCs	Disseminated tumour cells
EC	Endothelial cell
ECM	Extracellular matrix
EDG2	Lysophosphatidic acid receptor
ELEANORS	ESR1 locus enhancing and activating non-coding RNAs
EMT	Epithelial to mesenchymal transition
EphA5	Eph receptor 5
ER	Oestrogen receptor
FTIs	Farnesyl transferase inhibitors
HER2	Human epidermal growth factor receptor 2
IGF-1	Insulin growth factor-1
LncRNAs	Long non-coding RNAs
MALAT1	Metastasis-associated lung adenocarcinoma transcript 1
MHC	Major histocompatibility complex
miRNAs	Micro RNAs
MMC	Mouse mammary carcinoma
MMTV-PyMT	mouse mammary tumour virus-polyoma middle tumour-antigen
MRD	Minimal residual disease
NETs	Neutrophil extracellular traps
OS	Overall survival
Pfkfb3	6-phosphofructo-2-kinase/fructose-2,6-biphosphatase 3
PR	Progesterone receptor
PTX	Pentoxifylline
ROS	Reactive oxygen species
SIM	Simvastatin
TET	Ten-eleven translocation
TME	Tumour microenvironment
TNBC	Triple-negative breast cancer
TRAIL	TNF-alpha-Related Apoptosis-Inducing Ligand
TSP1	Thrombospondin-1
